# Public Psychosocial and Behavioral Responses in the First Wave of COVID-19 Pandemic: A Large Survey in China

**DOI:** 10.3389/fpsyt.2021.676914

**Published:** 2021-07-28

**Authors:** Huayu Yang, Xiaomeng Xian, Jing Hu, J. Michael Millis, Haitao Zhao, Xin Lu, Xinting Sang, Shouxian Zhong, Hui Zhang, Ping Yin, Yilei Mao

**Affiliations:** ^1^Department of Liver Surgery, Peking Union Medical College (PUMC) Hospital, PUMC & Chinese Academy of Medical Sciences (CAMS), Beijing, China; ^2^Department of Epidemiology and Health Statistics, School of Public Health, Tongji Medical College, Huazhong University of Science and Technology, Wuhan, China; ^3^Department of Surgery, Global Surgery, University of Chicago, Chicago, IL, United States; ^4^International Relations and Social Development Division, Horizon iDataWay, Beijing, China

**Keywords:** COVID-19, psychological impact, behavioral response, survey, epicenter

## Abstract

**Background:** The COVID-19 has grown into a global pandemic. This study investigated the public psychosocial and behavioral responses through different time periods of the pandemic, and assessed whether these changes are different in age, gender, and region.

**Methods:** A three-phase survey was conducted through the DaDui Social Q&A Software for COVID-19. A total of 13,214 effective responses of COVID-19 were collected. Statistical analysis was performed based on their basic information and psychosocial responses.

**Results:** The degree of attention, understanding, and cooperation with preventive and control measures of the disease increased and then decreased. The panic level gradually increased with the epidemic process. The degree of satisfaction with management measures and of confidence in defeating COVID-19 increased throughout the survey. Compared with residents in other areas, respondents from the COVID-19 epicenter (Wuhan) reported a higher degree of self-protection during the outbreak and a significantly lower degree of satisfaction with respect to government prevention and control measures during all phases. Shortages of medical supplies and low testing capacity were reported as the biggest shortcoming in the prevention and control strategies during COVID-19, and an abundance of disorderly and inaccurate information from different sources was the primary cause of panic.

**Conclusions and Relevance:** Major public health events elicit psychosocial and behavioral changes that reflect the different phases of the biologic curve. Sufficient medical supplies and improved organization and accurate information during epidemics may reduce panic and improve compliance with requested changes in behavior. We need to recognize this natural phenomenon and our public policy preparedness should attempt to move the social/psychological curve to the left in order to minimize and flatten the biologic curve.

## Introduction

At the beginning of 2020, the COVID-19 outbreak soon became a pandemic throughout the world, which generated vast public psychological and behavioral impacts apart from physical illness (1–4). Since the beginning of COVID-19, there have been several impactful studies related to the pathophysiology, treatment, and morbidity of COVID 19 (5–7). These studies are playing a key role in understanding and defeating the SARS CoV-2, but there has not been a sizable survey of the public's psychosocial and behavioral responses during the pandemic when we conducted this study. Such data would facilitate psychological assessments and interventions for COVID-19-related changes in mental health.

In order to isolate the coronavirus spreading and to prevent people from getting the disease, China had taken serious measurements in the affected areas and the Wuhan city was into a lockdown (8–10). Studies have shown that during the pandemic, people were more likely to suffer from emotional stresses, and the stresses may further affect certain social groups (11–14). Our study was designed to collect people's psychosocial changes to help us better understand these differences among different groups of people, so that we can assist their psychological needs in the future.

Here, we describe a large survey conducted throughout the development of the pandemic in China. We used the self-designed survey platform “DaDui Social Q&A Software” from Horizon iDataWay. Horizon iDataWay is a survey company with almost 30 years of experience in the investigation of social events with good reputations. The data from their company have been used and cited numerous times in international scientific publications and news outlets (also see Supplementary Table 1) (15). In this study, 13,214 responses regarding the COVID-19 were collected from participants in 31 provinces and municipalities in China. The survey collected respondents' basic information, disease awareness, behavioral responses, and psychosocial effects of this pandemic. We analyzed the data to identify psychosocial and behavioral changes associated with the COVID-19 pandemic.

The purpose of the study was to examine the self-reported psychosocial and behavioral changes during different stages of a major public health emergency. We also studied the different responses between age, gender, geographic region (severity of the pandemic), which provided valuable information for future studies and prevention.

## Methods

### Survey Tools

The COVID-19 survey was conducted using DaDui Social Q&A Software (Beijing-based consultancy Horizon iDataWay, Registration No. 2019SR1335251). The design of the COVID-19 survey followed the guidelines of the European Society for Opinion and Marketing Research (ESOMAR), and a database management system was established in accordance with ESOMAR requirements to protect the privacy of respondents.

The inclusion criteria required participants aged between 18 and 70 and were Chinese citizens during the pandemic. Participants were informed that the survey data would be collected for research analyses. After they agreed to participate in this survey, the eligible respondents could be accessed to the survey through their cellphone.

The survey was based on a software platform that can guarantee data quality through the following exclusion criteria: (1) The system background automatically recorded the answer time, and responses with abnormal response times (too short or too long) were excluded. (2) The answer logic was set in advance; if an answer failed to meet the logic, the survey could not be continued. (3) A “lie-detector question” was included; if the answers were inconsistent before and after, the response was excluded.

This was a voluntary online survey, and the respondents did not receive guidance when answering the survey questions. The specific questions included in the survey are shown in Supplementary Table 2. There were 12 questions that collected basic personal information, disease awareness, personal behavioral responses, and psychosocial effects.

### COVID-19 Survey Population and Survey Duration

The three COVID-19 surveys were monitored using the interactive survey system created by Horizon iDataWay. The epidemic curve of confirmed COVID-19 cases in China showed that: sporadic cases of unknown caused pneumonia appeared in Wuhan in January; newly confirmed cases of COVID-19 peaked in February; the cases slowly declined and the coronavirus was nearly eradicated in China in April. The first wave of the COVID-19 pandemic in China was from January (Wuhan city in lockdown on January 23rd, 2020) to April 2020 (Wuhan lockdown ended on April 8th, 2020). The three surveys in this study represented the key episodes of the first wave of COVID-19 in China, which include the early stage of the COVID-19 pandemic in Wuhan (cluster of sporadic new cases, January 25th−30th, 2020), the outbreak stage with exponentially growing new cases (February 4th−7th, 2020), and the late stage (confirmed cases decreasing, February 22nd−27th, 2020) to demonstrate public psychosocial and behavioral changes. Survey I covered 30 provinces and municipalities (except Tibet) with an effective sample size of 1,674. Survey II covered 31 provinces and municipalities with an effective sample size of 6,685. Survey III covered 31 provinces and municipalities with an effective sample size of 4,855. After Survey I, 9,692 cases were confirmed in China with 213 deaths. After Survey II, 34,546 cases were confirmed with 722 deaths. After Survey III, 78,824 cases were confirmed with 2,788 deaths.

### Statistical Analysis

The results of the descriptive analyses are presented as means (standard deviations, SDs) or percentages (%). Unless otherwise indicated, we used either a single factor analysis of variance (ANOVA) test or a Chi-square test to compare differences between subgroups based on the key periods of the first wave of the pandemic and participant characteristics. Bonferroni's correction was used to reduce the probability of a type I error when multiple testing. All statistical analyses were performed using SPSS software (version 23.0) with a two-sided significance threshold of *P* < 0.05.

## Results

### COVID-19 Survey Results

**Figure 1:**
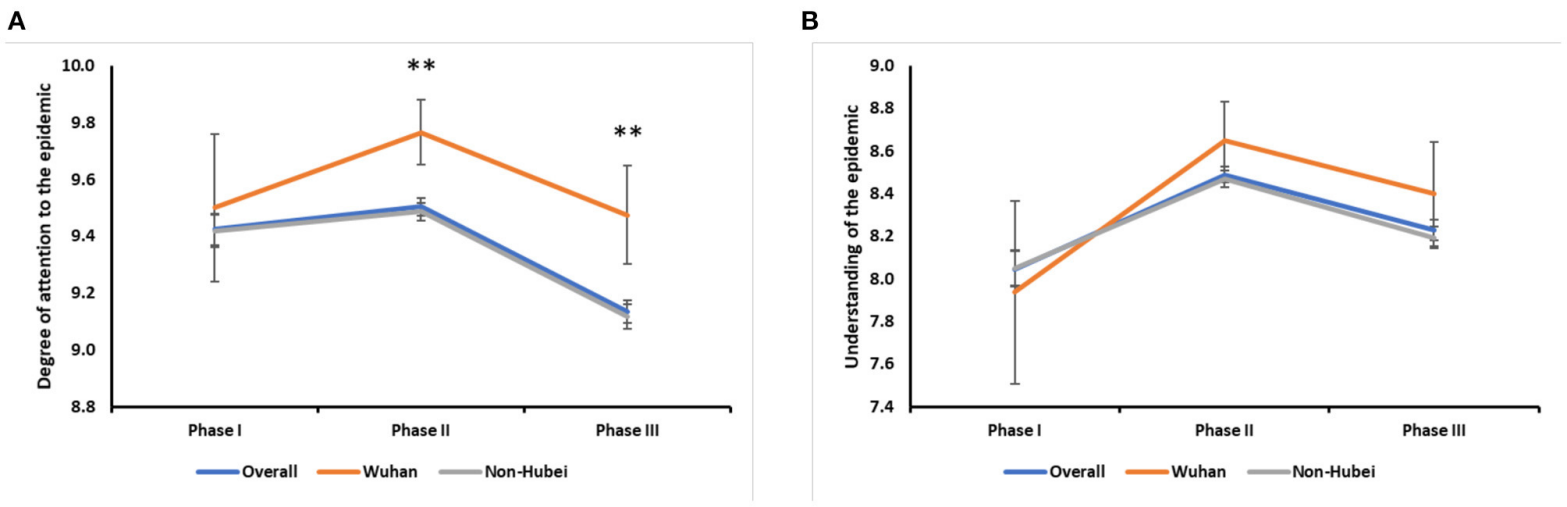
The disease awareness of COVID-19. **(A)** Changes in the degree of attention to the epidemic in overall and different areas: overall (^**^:I vs. III, *P* < 0.001; II vs. III, *P* < 0.001); Wuhan (II vs. III, *P* = 0.016); Non-Hubei (I vs. III, *P* < 0.001; II vs. III, *P* < 0.001). Survey II (Wuhan vs. Non-Hubei, *P* < 0.001); Survey III (Wuhan vs. Non-Hubei, *P* < 0.001). **(B)** Changes in the degree of understanding in overall and different areas: overall (I vs. II, *P* < 0.001; I vs. III, *P* < 0.001; II vs. III, *P* < 0.001); Wuhan (I vs. II, *P* = 0.004); Non-Hubei (I vs. II, *P* < 0.001; I vs. III, *P* = 0.008; II vs. III, *P* < 0.001).

**Figure 2:**
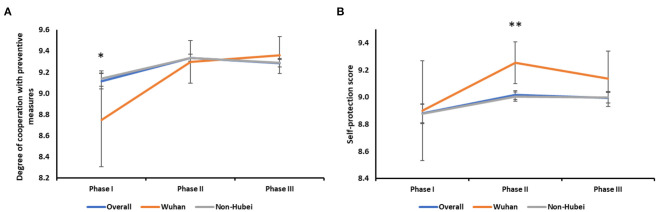
Personal behavioral responses to COVID-19. **(A)** Changes in the degree of cooperation in overall and different areas: overall (*:I vs. II, *P* < 0.001; I vs. III, *P* < 0.001); Wuhan (I vs. II, *P* = 0.023; I vs. III, *P* = 0.010); Non-Hubei (I vs. II, *P* < 0.001; I vs. III, *P* < 0.001). Survey I (Wuhan vs. Non-Hubei, *P* = 0.025). **(B)** Changes in the degree of self-protection in overall and different areas: overall (I vs. II, *P* = 0.001; I vs. III, *P* = 0.010); Non-Hubei (I vs. II, *P* = 0.003; I vs. III, *P* = 0.008). Survey II (**:Wuhan vs. Non-Hubei, *P* = 0.003).

**Figure 3:**
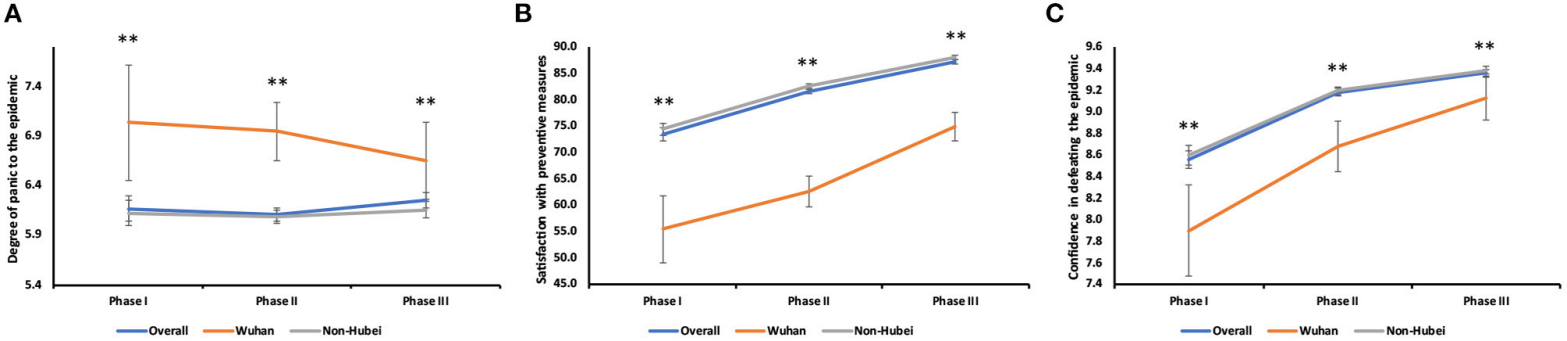
Psychosocial impact from COVID-19. **(A)** Changes in panic level in overall and different areas: overall (II vs. III, *P* = 0.013). Survey I (**:Wuhan vs. Non-Hubei, *P* = 0.002); Survey II (**:Wuhan vs. Non-Hubei, *P* < 0.001); Survey III (**:Wuhan vs. Non-Hubei, *P* = 0.009). **(B)** Changes in the degree of satisfaction in overall and different areas: overall (I vs. II, *P* < 0.001; I vs. III, *P* < 0.001; II vs. III, *P* < 0.001); Wuhan (I vs. II, *P* = 0.049; I vs. III, *P* < 0.001; II vs. III, *P* < 0.001); Non-Hubei (I vs. II, *P* < 0.001; I vs. III, *P* < 0.001; II vs. III, *P* < 0.001). Survey I (**:Wuhan vs. Non-Hubei, *P* < 0.001); Survey II (**:Wuhan vs. Non-Hubei, *P* < 0.001); Survey III (**:Wuhan vs. Non-Hubei, *P* < 0.001). **(C)** Changes in the level of confidence in overall and different areas: overall (I vs. II, *P* < 0.001; I vs. III, *P* < 0.001; II vs. III, *P* < 0.001); Wuhan (I vs. II, *P* = 0.002; I vs. III, *P* < 0.001; II vs. III, *P* = 0.018); Non-Hubei (I vs. II, *P* < 0.001; I vs. III, *P* < 0.001; II vs. III, *P* < 0.001). Survey I (**:Wuhan vs. Non-Hubei, *P* = 0.001); Survey II (**:Wuhan vs. Non-Hubei, *P* < 0.001); Survey III (**:Wuhan vs. Non-Hubei, *P* = 0.003).

In this study, the COVID-19 Survey I involved a total of 1,674 people (male 30.8%, age 34.1 ± 10.1), Survey II involved 6,685 people (male 37.5%, age 34.4 ± 10.7), and Survey III involved 4,855 people (male 39.1%, age 30.1 ± 10.8). We calculated the average scores for all questions and the results were plotted in Figures 1–3 and Supplementary Table 3.

#### Disease Awareness

In terms of disease awareness, respondents increased their level of understanding and focus on the COVID-19 epidemic between Survey I and II of the study. The degree of attention was highest in Survey II, reaching 9.50 ± 1.24 (out of 10), but that significantly declined in Survey III (*p* < 0.001) (Figure 1A). The degrees of attention reported by non-Hubei residents were consistent with the overall scores. The scores of Wuhan residents also peaked in Survey II, but only the scores of Surveys II and III significantly differed (*P* = 0.016), not Survey I. As reported, Wuhan residents had a significantly higher degree of attention paid to the epidemic in Surveys II and III in comparison with non-Hubei residents (*P* < 0.001, *P* < 0.001), though both Wuhan and non-Hubei residents paid the equal attention to the epidemic in Survey I. In Survey III, while the degree of attention in the overall case was decreased, Wuhan residents maintained a high level of attention with respect to the epidemic.

The degree of understanding regarding the epidemic was also highest in Survey II, reaching 8.49 ± 1.55 (out of 10) (Figure 1B). For non-Hubei residents, the trend was similar to the overall trend, and all Surveys had significantly different scores (Survey I vs. II, *P* < 0.001; I vs. III, *P* = 0.008; II vs. III, *P* < 0.001). Within Wuhan residents, the scores were only significantly differed between Surveys I and II (*P* = 0.004). During Survey I, Wuhan residents had the lower level of understanding than non-Hubei residents regarding the disease, but their level of understanding exceeded that of non-Hubei residents in Surveys II and III. However, these differences were not significant.

#### Personal Behavior

In terms of personal behavior, the degree of cooperation with government prevention and control measures was lowest in Survey I, especially among Wuhan residents (8.75 ± 2.02, *p* = 0.025). The score increased in Survey II and then remained relatively constant in Survey III (Figure 2A). The overall degree of self-protection was highest at Survey II (9.02 ± 1.33) and slightly decreased in Survey III (Figure 2B). The score in non-Hubei residents was consistent with the overall score (I vs. II, *P* = 0.003; I vs. III, *P* = 0.008). The self-protection scores of the three surveys in Wuhan residents had no significant difference. It was worth to mention that in Survey II, the degree of self-protection reported for Wuhan residents was significantly higher than that reported for non-Hubei residents (*P* = 0.003).

#### Psychosocial Impact

In terms of psychosocial impact, the scores reflecting levels of panic in respondents were relatively constant, with a slight decline in Survey II and a slight rebound in Survey III (II vs. III, *P* = 0.013) (Figure 3A). There were significant differences between Wuhan residents and non-Hubei residents (I, *P* = 0.002; II, *P* < 0.001; III, *P* = 0.009) for comparing the panic levels. Specifically, in Survey I, Wuhan residents had the higher degree of panic than non-Hubei residents, but their panic level continued to decrease over time. The overall degree of satisfaction with the government's prevention and control measures gradually increased with time, reaching 87.28 ± 15.25 (out of 100) in Survey III (Figure 3B). The satisfaction scores of Wuhan residents showed the increasing trend, and the changes in increases were significant in all three surveys (I vs. II, *P* = 0.049; I vs. III, *P* < 0.001; II vs. III, *P* < 0.001). In Surveys I and II, the satisfaction scores of Wuhan citizens were 25% lower than those of non-Hubei residents. In Survey III, this gap decreased to 15%, and the differences were significant in all three surveys (All *P* < 0.001). The overall levels of confidence in defeating the epidemic also gradually increased over time (Figure 3C). The confidence scores of non-Hubei residents were significantly higher than those of Wuhan residents in all surveys (I, *P* = 0.001; II, *P* < 0.001; III, *P* = 0.003).

### Stratified Analysis of Gender and Age in COVID-19 Survey Respondents

**Figure 4:**
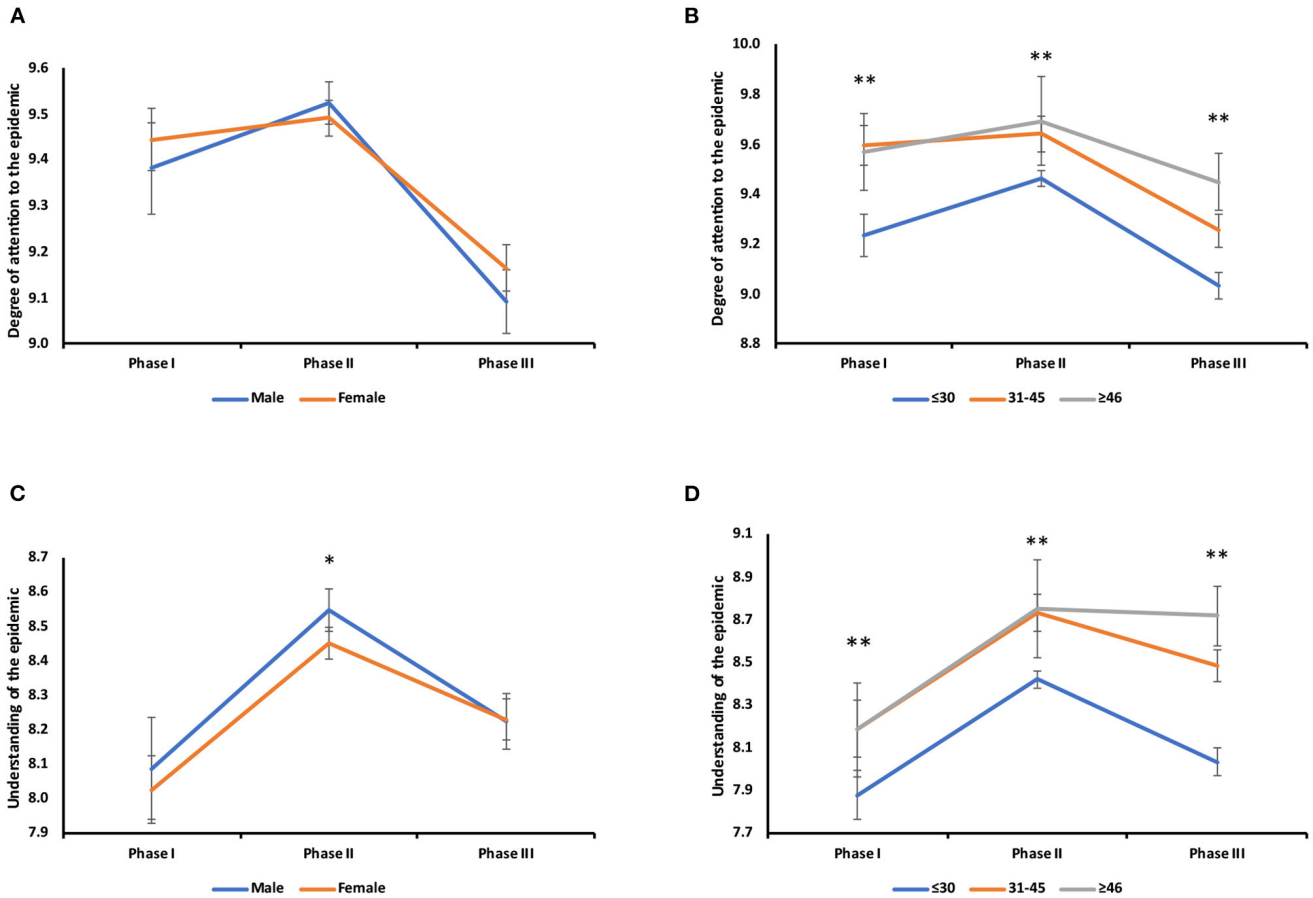
Gender and age stratified analysis of the disease awareness of COVID-19. **(A)** Changes in the degree of attention to the epidemic in different genders: male (I vs. III, *P* < 0.001; II vs. III, *P* < 0.001), female (I vs. III, *P* < 0.001; II vs. III, *P* < 0.001). **(B)** Changes in the degree of attention to the epidemic in different age groups: under 30 years old: I vs. II, *P* < 0.001; I vs. III, *P* = 0.001; II vs. III, *P* < 0.001), 31–45 years old (I vs. III, *P* < 0.001; II vs. III, *P* < 0.001), above 46 years old (I vs. II, *P* = 0.001; I vs. III, *P* < 0.001). Survey I (**:≤30 vs. 31–45, *P* < 0.001; ≤30 vs. ≥46, *P* < 0.001), Survey II (**:≤30 vs. 31–45, *P* < 0.001; ≤30 vs. ≥46, *P* = 0.013) Survey III (**:≤30 vs. 31–45, *P* < 0.001; ≤30 vs. ≥46, *P* < 0.001; 31–45 vs. ≥46, *P* = 0.034). **(C)** Changes in the degree of understanding in different genders: male (*:I vs. II, *P* < 0.001; II vs. III, *P* < 0.001), female (*:I vs. II, *P* < 0.001; I vs. III, *P* = 0.001; II vs. III, *P* < 0.001). Survey II (*P* = 0.014). **(D)** Changes in the degree of understanding in different age groups: under 30 years old (I vs. II, *P* < 0.001; II vs. III, *P* < 0.001). Survey I (**:≤30 vs. 31–45, *P* = 0.002; ≤30 vs. ≥46, *P* = 0.032), Survey II (**:≤30 vs. 31–45, *P* < 0.001; ≤30 vs. ≥46, *P* = 0.041), Survey III (**:≤30 vs. 31–45, *P* < 0.001; ≤30 vs. ≥46, *P* < 0.001; 31–45 vs. ≥46, *P* = 0.026).

We conducted a stratified analysis of scores from the three surveys based on gender and age. The scores of disease awareness all peaked in Survey II (Figures 4A–D). In terms of the degree of attention paid to the epidemic, there was no significant difference between men and women (Figure 4A). However, it was significantly different between age groups (Figure 4B). In terms of the level of understanding of the disease, there were significant differences in both gender and age. However, the difference between men and women was only significant in Survey II (Figure 4C). Respondents who were older than 31 years had a significantly higher degree of understanding regarding the epidemic than those under 31 years in the three surveys (Figure 4D).

**Figure 5:**
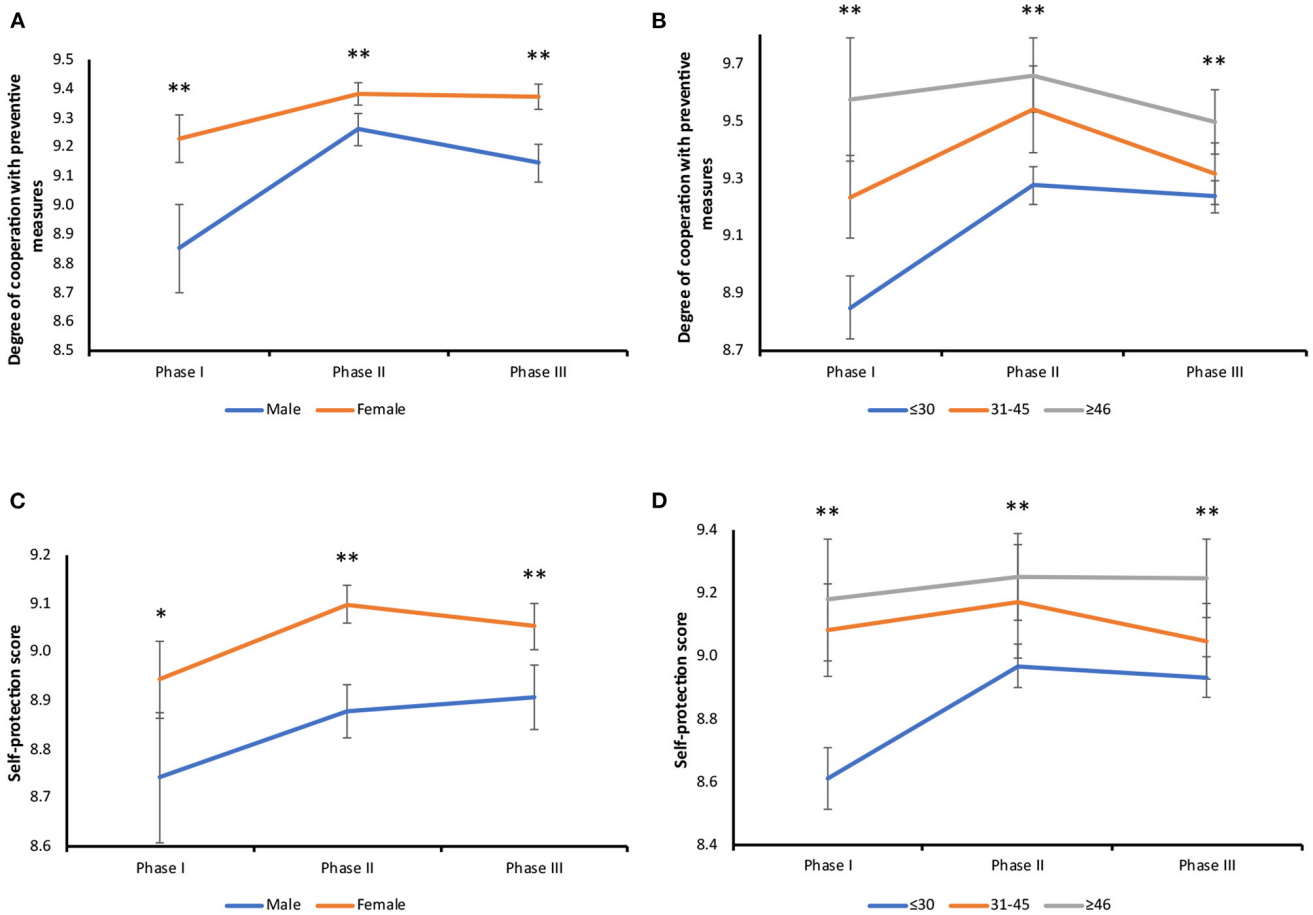
Gender and age stratified analysis of personal behavioral responses to COVID-19. **(A)** Changes in the degree of cooperation in different genders: male (I vs. II, *P* < 0.001; I vs. III, *P* < 0.001; II vs. III, *P* = 0.029), female (I vs. II, *P* = 0.001; I vs. III, *P* = 0.003). Survey I (**:*P* < 0.001), Survey II (**:*P* < 0.001), Survey III (**:*P* < 0.001). **(B)** Changes in the degree of cooperation in different age groups: under 30 years old (I vs. II, *P* < 0.001; I vs. III, *P* = 0.001), 31–45 years old (I vs. II, *P* < 0.001; II vs. III, *P* < 0.001). Survey I (**:≤30 vs. 31–45, *P* < 0.001; ≤30 vs. ≥46, *P* < 0.001; 31–45 vs. ≥46, *P* = 0.006), Survey II (**:≤30 vs. 31–45, *P* < 0.001; ≤30 vs. ≥46, *P* < 0.001), Survey III (**:≤30 vs. ≥46, *P* < 0.001; 31–45 vs. ≥46, *P* = 0.027). **(C)** Changes in the degree of self-protection in different genders: female (I vs. II, *P* = 0.001; I vs. III, *P* = 0.049). Survey I (*P* = 0.009), Survey II (**:*P* < 0.001), Survey III (**:*P* < 0.001). **(D)** Changes in the degree of self-protection in different age groups: under 30 years old (I vs. II, *P* < 0.001; I vs. III, *P* < 0.001), 31–45 years old (II vs. III, *P* = 0.027). Survey I (**:≤30 vs. 31–45, *P* = 0.002; ≤30 vs. ≥46, *P* = 0.032), Survey II (**:≤30 vs. 31–45, *P* < 0.001; ≤30 vs. ≥46, *P* = 0.014), Survey III (≤30 vs. 31–45, *P* = 0.037; **:≤30 vs. ≥46, *P* < 0.001; 31–45 vs. ≥46, *P* = 0.025).

The degree of cooperation with government prevention and control measures, women reported a significantly higher level of cooperation including self-protection than men in all surveys (Figures 5A,B). All age groups had their highest degree of cooperation in Survey II, and participants older than 45 had the significantly highest level of cooperation than the other two age groups in all three surveys (Figure 5C). Similarly, the age group above 45 years also had the significantly highest level of self-protection in all three surveys (Figure 5D).

**Figure 6:**
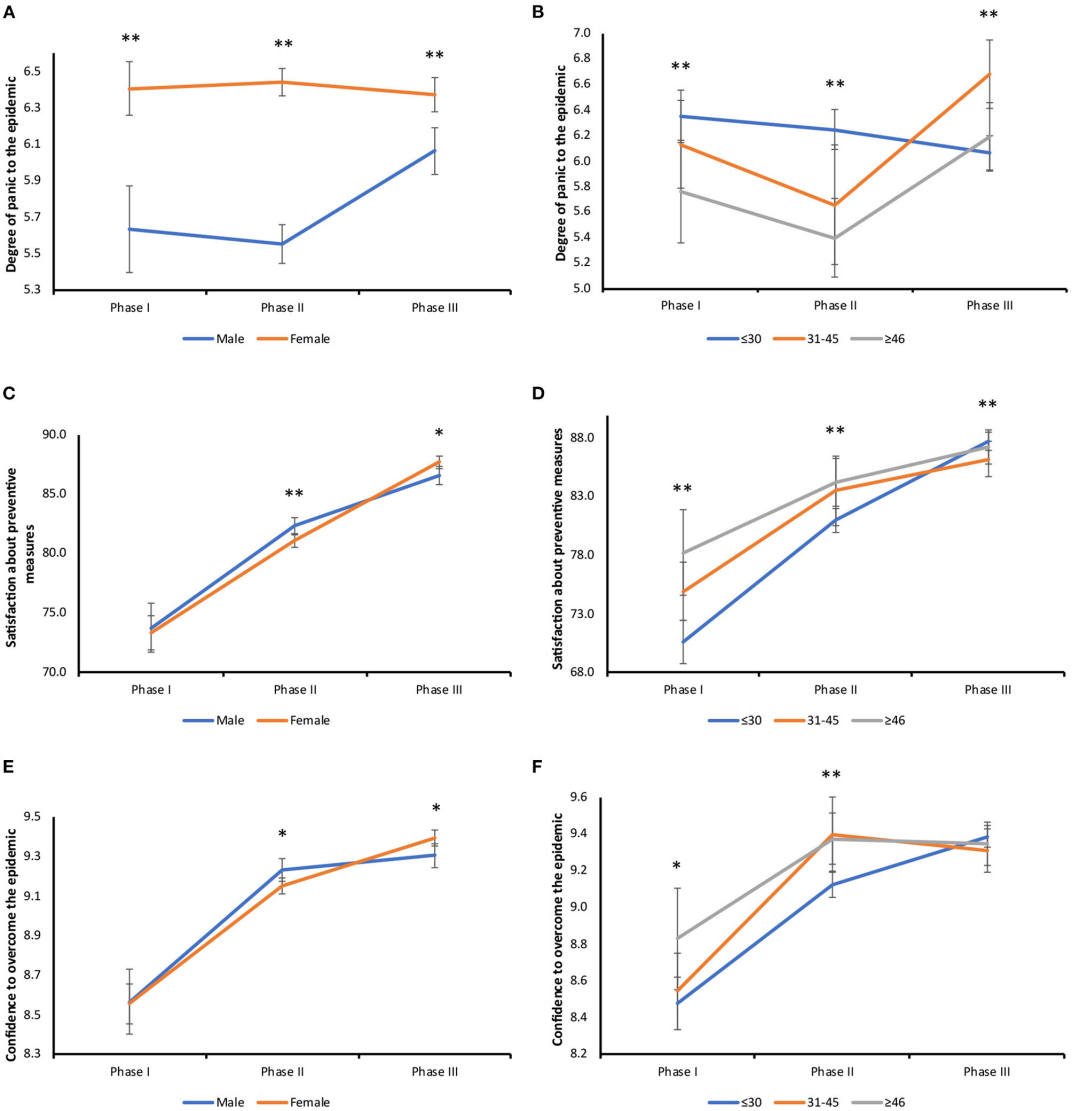
Gender and age stratified analysis of psychosocial effects from COVID-19. **(A)** Changes in panic level in different genders: male (I vs. III, *P* = 0.006; II vs. III, *P* < 0.001). Survey I (**:*P* < 0.001), Survey II (**:*P* < 0.001), Survey III (**:*P* < 0.001). **(B)** Changes in panic level in different age groups: under 30 years old (I vs. III, *P* = 0.022; II vs. III, *P* = 0.008), 31–45 years old (I vs. II, *P* = 0.001; I vs. III, *P* < 0.001; II vs. III, *P* < 0.001), above 46 years old (II vs. III, *P* = 0.007). Survey I (**:≤30 vs. ≥46, *P* = 0.005), Survey II (**:≤30 vs. 31–45, *P* < 0.001;≤30 vs. ≥46, *P* < 0.001), Survey III (**:≤30 vs. 31–45, *P* < 0.001; 31–45 vs. ≥46, *P* = 0.002). **(C)** Changes in the degree of satisfaction in different genders: male (*:I vs. II, *P* < 0.001; I vs. III, *P* < 0.001; II vs. III, *P* < 0.001), female (*:I vs. II, *P* < 0.001; I vs. III, *P* < 0.001; II vs. III, *P* < 0.001). Survey II (*P* = 0.006), Survey III (*P* = 0.010). **(D)** Changes in the degree of satisfaction in different age groups: under 30 years old (I vs. II, *P* < 0.001; I vs. III, *P* < 0.001; II vs. III, *P* < 0.001), 31–45 years old (I vs. II, *P* < 0.001; I vs. III, *P* < 0.001; II vs. III, *P* = 0.001), above 46 years old (I vs. II, *P* = 0.003; I vs. III, *P* < 0.001). Survey I (**:≤30 vs. 31–45, *P* = 0.003;≤30 vs. ≥46, *P* < 0.001), Survey II (**:≤30 vs. 31–45, *P* < 0.001), Survey III (**:≤30 vs. 31–45, *P* = 0.005). **(E)** Changes in the level of confidence in different genders: male (*:I vs. II, *P* < 0.001; I vs. III, *P* < 0.001), female (*:I vs. II, *P* = 0.001; I vs. III, *P* = 0.049; II vs. III, *P* < 0.001). Survey II (*P* = 0.024), Survey III (*P* = 0.017). **(F)** Changes in the level of confidence in different age groups: under 30 years old (*:I vs. II, *P* < 0.001; I vs. III, *P* < 0.001; II vs. III, *P* < 0.001), 31–45 years old (I vs. II, *P* < 0.001; I vs. III, *P* < 0.001), above 46 years old (I vs. II, *P* < 0.001; I vs. III, *P* < 0.001). Survey I (≤30 vs. ≥46, *P* = 0.018), Survey II (**:≤30 vs. 31–45, *P* < 0.001).

About the psychosocial effect, women had a constantly higher level of panic than that in men. Men reported a relatively low level of panic in Survey I and II, but the score significantly increased in Survey III (Figure 6A). Participants in different age groups also reported significantly different degrees of panic. Those in the age group below 31 showed a continuous decrease in panic level, reaching its lowest in Survey III, while those above 30 years reported the lowest degree of panic in Survey II, bouncing back in Survey III. In Surveys I and II, the participants below 31 had the highest level of panic than the other two age groups, but their panic level became the lowest among three groups in Survey III (Figure 6B). In terms of satisfaction with government prevention and control measures, both men and women reported a significant increase throughout the three surveys (Figure 6C). Similarly, in the age groups, the level of satisfaction also continued to increase significantly. Participants younger than 31 had the lowest level of satisfaction in Survey I and II, but their level of satisfaction became the highest among all age groups in Survey III (Figure 6D). In terms of confidence in defeating the epidemic, both men and women had an increased level of confidence through the three-survey periods (Figure 6E). Respondents below 31 also had the most drastic changes in their level of confidence throughout the three surveys. Their level of confidence was significantly lower compared to the other two age groups in Survey I and II, but they had the highest level of confidence in Survey III (Figure 6F).

### Shortcomings of the Precautional Measures

**Figure 7:**
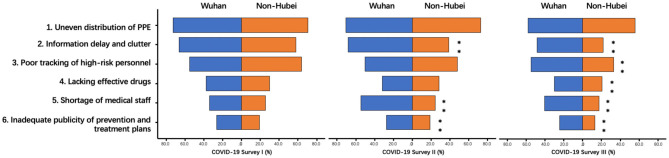
Shortcomings of prevention and control measures during COVID-19. **Indicates significant differences between Wuhan vs Non-Hubei residents.

The respondents were asked to report what they felt to be shortcomings regarding the prevention and control measures. Table 1 contains the six given options. As shown in Figure 7, 70.9% of the people selected “uneven distribution of personal protective equipment (PPE),” making it the biggest shortcoming in all surveys. However, its proportion significantly decreased over time (both *p* < 0.001). Other shortcomings selected by Wuhan residents were also shown, with “information delay and clutter” being the second biggest shortcoming. Among non-Hubei residents, “poor tracking of high-risk (migrant) personnel” was the second biggest shortcoming instead.

**Table 1:** Shortcomings of precautionary measures and causes of panic (in percentage).

	**Survey I**		**Survey II**		**Survey III**		***P*-value**
	***N* = 1,674**	**%**	***N* = 6,685**	**%**	***N* = 4,855**	**%**	
**Shortcomings of prevention and control measures**							
1. Uneven distribution of PPEs	1,187	70.9	4,814	72.0	2,686	55.3	<0.001
2. Poor tracking of high-risk personnel	1,059	63.3	3,220	48.2	1,623	33.4	<0.001
3. Information delay and clutter	974	58.2	2,677	40.0	1,150	23.7	<0.001
4. No effective drugs against COVID-19	507	30.3	1,900	28.4	1,061	21.9	<0.001
5. Medical staff short-handed	434	25.9	1,772	26.5	936	19.3	<0.001
6. Inadequate publicity of prevention and treatment plans	324	19.4	1,287	19.3	675	13.9	<0.001
**Causes of panic**							
1. Too much disorderly and cluttered information from different source	876	52.3	3,865	57.8	2,425	49.9	<0.001
2. Masks and sanitizers sold out	787	47.0	3,554	53.2	2,308	47.5	<0.001
3. Migrant workers from Other/Hubei Province	486	29.0	1,465	21.9	896	18.5	<0.001
4. Family members not taking the disease seriously	411	24.6	936	14.0	630	13.0	<0.001
5. Being contact with people from Hubei Province/I live in Wuhan	114	6.8	461	6.9	239	4.9	<0.001

*Shortcomings of prevention and control measures: option 1: I vs. III, P < 0.001, II vs. III, P < 0.001; option 2: I vs. II, P < 0.001, I vs. III, P < 0.001, II vs. III, P < 0.001; option 3: I vs. II, P < 0.001, I vs. III, P < 0.001, II vs. III, P < 0.001; option 4: I vs. III, P < 0.001, II vs. III, P < 0.001; option 5: I vs. III, P < 0.001, II vs. III, P < 0.001; option 6: I vs. III, P < 0.001, II vs. III, P < 0.001*.

*Causes of panic: option 1: I vs. II, P < 0.001, II vs. III, P < 0.001; option 2: I vs. II, P < 0.001, II vs. III, P < 0.001; option 3: I vs. II, P < 0.001, I vs. III, P < 0.001, II vs. III, P < 0.001; option 4: I vs. II, P < 0.001, I vs. III, P < 0.001; option 5: I vs. III, P = 0.003, II vs. III, P < 0.001*.

In Survey II, the two biggest shortcomings chosen by Wuhan residents were still the same as in Survey I, but “shortage of medical staff” became the third, while it ranked fifth in Survey I. The ranking of options remained the same among non-Hubei residents in Survey II. In Survey II, the percentages of respondents who chose “information delay and clutter,” “shortage of medical staff,” and “inadequate publicity regarding prevention and treatment plans” were significantly different between Wuhan residents and non-Hubei residents (*p* < 0.001; *p* < 0.001; *p* = 0.001).

In Survey III, Wuhan residents still chose “uneven distribution of PPE” as the biggest shortcoming, followed by “poor tracking of high-risk personnel,” “information delay and clutter,” “shortage of medical staff,” and the last two remained the same. Non-Hubei residents gave similar rankings in Surveys I and II. In Survey III, the percentages of respondents who chose “information delay and clutter,” “poor tracking of high-risk personnel,” “lack of drugs effective against COVID-19,” “shortage of medical staff,” and “inadequate publicity of prevention and treatment plans” were significantly different between Wuhan residents and non-Hubei residents (*P* < 0.001; *p* < 0.001; *p* = 0.001; *P* < 0.001; *P* < 0.001).

In all three surveys, the percentages of Wuhan residents who chose each option were greater than those of non-Hubei residents. In the sub-comparison of the three surveys, the proportion of Wuhan citizens who chose “uneven distribution of PPE” significantly decreased over time (Survey I vs Survey II, *p* = 0.021, Survey II vs. Survey III, *p* = 0.004) while the proportion of Wuhan citizens who chose “information delay and clutter” peaked in Survey II (Survey I vs. Survey II, *p* = 0.006, Survey II vs. Survey III, *P* < 0.001).

Among the non-Hubei residents, the proportions of respondents who chose “poor tracking of high-risk personnel” and “information delay and clutter” significantly decreased in Survey II compared with Survey I (both *p* < 0.001). The proportion of all five shortcomings reported by non-Hubei residents were significantly decreased from Survey I to Survey III (all *p* < 0.001).

### Causes of Panic

**Figure 8:**
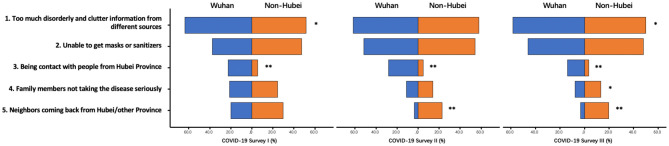
Causes of panic during COVID-19. **indicates significant differences between Wuhan and Non-Hubei. *indicates significant difference among three surveys.

The respondents were also asked to select the causes of feelings of panic. Five options were given, as shown in Table 1. The proportions of each option selected by respondents were plotted in Figure 8. Overall, the proportion of respondents who selected “too much disorderly and cluttered information from different sources,” “masks and sanitizers sold out,” and “being in contact with people from Hubei Province/I live in Wuhan” peaked in Survey II (all *p* < 0.001). The proportion of respondents who chose the remaining two options continued to decrease.

The ranking of causes of panic remained the same among Wuhan residents in all three surveys. Specifically, the top choice “too much disorderly and cluttered information” was relatively constant. The second one “masks and sanitizers sold out” significantly increased in Survey II (*p* = 0.028). The third one “I live in Wuhan” was significantly lowered in Survey III (*p* < 0.001). Among the non-Hubei residents, the rankings of the causes of panic remained the same through all three surveys. The first two were identical to those chosen by Wuhan residents. The third was “neighbors come from Hubei/other provinces,” followed by “family members not taking the disease seriously,” and “being in contact with people from Hubei Province.” In Survey I, the proportions of respondents who selected “too much disorderly and cluttered information” significantly differed between Wuhan residents and non-Hubei residents (*p* = 0.037). In Survey III, except for “masks and sanitizers sold out,” the proportions of respondents who selected other options significantly differed between Wuhan residents and non-Hubei residents.

## Discussion

The SARS epidemic in 2003 was the first global public health emergency in the twenty-first century. Many countries have realized that because of increased international travel and trade across the globe, public health security is no longer localized in a single country or region, but global cooperation is needed. Since 2003, several other epidemics or pandemics have occurred, including the H1N1 pandemic in 2009 (16, 17), the polio surge in May 2014, the Ebola outbreak in West Africa in August 2014, the Zika virus epidemic in 2016, and the Ebola outbreak in Congo in 2019. Each outbreak was a huge threat to public health. The world needs to work together to fight against each epidemic (18).

In this study, we received 13,214 responses regarding the COVID-19 from individuals in 31 provinces and municipalities in mainland China. The data indicate that during the epidemic, the public's level of attention regarding the disease was high initially, with minimal regional differences. As time progressed, the level of attention reached a peak in mid-February when both traditional media and social media started to release the numerous reports about the epidemic. In late-February, COVID-19 was basically under control in non-Hubei areas. At this point, the level of attention to the epidemic decreased significantly in non-Hubei residents, while Wuhan residents still maintained a high level of attention. There were no significant differences in the degree of understanding regarding the disease by region, which suggests that people in different regions of China have equal access to information about the disease. In terms of behavioral responses, in late January, when cluster of new COVID-19 cases popped up, respondents initially reported a lower degree of cooperation with prevention and control measures. However, their level of cooperation gradually increased as the epidemic progressed. Also, respondents who lived in the epicenter of the outbreak engaged in higher levels of preventative behaviors. This finding was as expected because people in the epicenter were at a higher risk of getting the virus. In terms of psychosocial changes, we found significant differences between respondents in different regions. Wuhan residents reported a lower degree of satisfaction with the government's prevention and control measures in the early stage of the epidemic. This may have been due to initial chaos and confusion, insufficient medical supplies, and inadequate organization and coordination. As time progressed, the level of satisfaction with preventative measures in Wuhan residents gradually increased, their level of panic gradually decreased, and their confidence in defeating the epidemic gradually increased. In fact, by conducting strict lockdown orders, people may feel less psychological stress because their physical distance to the virus increased (19). These psychosocial changes were consistent with the score of Wuhan residents and the impressions reported by the media. Another study about the social isolation in Italy showed that longer isolation could increase people's mental stress (20). Government needs to take these factors into account when they implement social distancing policies and provide adequate online mental support for people in need.

The present data indicate that gender and age were important factors that affected individual psychological and behavioral changes during the epidemic. The degree of attention paid to the disease, cooperation with prevention and control measures, degree of self-protection, and panic level were all higher in older people compared with younger people. Interestingly, in the early stage of the epidemic, older people had a higher degree of satisfaction with the prevention and control measures and greater confidence in defeating the epidemic, while in the later stage of the epidemic, these levels significantly decreased, which is the opposite of the survey results from the younger population. It is possible that the younger people realized they had less risk of dying and thus their confidence in surviving the epidemic increased and thus more satisfied with the prevention and control measures whereas the elderly people still felt vulnerable than thus still dissatisfied with the prevention and control measures.

The data indicated that the level of panic was maintained at a relatively high level among female respondents. Two large studies conducted in Bangladesh and Philippine confirmed our finding, in which women were more likely to be suffered from psychological issues from the pandemic (21, 22). This continuous feeling of panic may have motivated women to maintain a higher degree of self-protection and to be more cooperative with the prevention and control measures compared with men throughout the first wave of the epidemic (23). One possible explanation for this result was that those Chinese women, who are usually the decision-makers of their families, may have prompted their family members to take the disease seriously, and to engage in preventative measures, such as reducing outdoor activities and using personal protection.

The results showed that the biggest perceived shortcoming of the prevention and control measures during the outbreak was the shortage of medical supplies. Additionally, in the early stage of the epidemic in the highest-risk area (Wuhan), the information delay and clutter, low testing capacities, and shortages of medical staff were also big issues. These issues were significantly less frequently rated as shortcomings in the later stage of the epidemic. This is likely a response to a national medical supply reallocation project and an influx of medical workers from other provinces of China to Wuhan and other cities in Hubei Province to help fight COVID-19.

The data regarding the causes of panic showed that during the entire COVID-19 epidemic, the top cause of panic was the abundance of disorder and inaccurate information from different sources. This indicates that an inability to determine the authenticity of information was the biggest cause of panic among the respondents, especially the respondents who lived in Wuhan. This finding is consistent with the viewpoint that media hype can cause panic, as mentioned by Ippolito et al. (24). The resolution of this issue is more difficult in the current age of social media whereas many people, especially younger people, place the same validity on a single social media post as validated scientific information. Of course, when dealing with a new disease it is easy to draw incorrect conclusions even from validated scientific data. Overall, these data indicate that in the current fast-developing world, it is very important to release accurate and authoritative information promptly. Furthermore, adequate medical supplies may play an important role in reducing panic among the public. In addition, people's psychological changes are also associated with their physical health. A recent study showed that the increased level of anxiety had resulted in a greater chance of obesity (25). All governments should keep these in mind so they can address similar future epidemics in a more focused and effective manner.

In this study, we compared the individual psychological changes caused by two major public health events that occurred 17 years apart. We reviewed the results of a telephone survey during the SARS epidemic 17 years ago (SARS data not published). Although these two surveys had multiple different variables (sample size, epicenter, method, and platform) that made them statistically incomparable, we tried to make a presentative comparison of the two, hoping to provide some valuable information. The degree to which respondents engaged in coordination and self-protection with respect to prevention and control measures was lower during SARS than COVID-19, suggesting that after experiencing the SARS epidemic in 2003, people of the same generation had experiences in dealing with an epidemic that led them to significantly improve their degree of cooperation with the government prevention and control measures during the COVID-19 pandemic. The degree of self-protection also increased. Satisfaction regarding the prevention and control measures and confidence with respect to defeating the epidemic in the later period of COVID-19 generally appeared to be higher than the corresponding data from the late period of SARS. One possible explanation was that Chinese citizens generally had a positive view of government under the influence of China's rapid economic development over the past decade. Satisfaction and confidence scores varied according to age in the later period of both epidemics. Younger people had higher satisfaction and confidence scores in the late stage of the COVID-19 epidemic, while this group had lower scores in the late stage of the SARS epidemic. We considered that this was because younger people who could actively participate in the current rapid development of Chinese society were more optimistic and had a higher degree of recognition with respect to the country, while these sentiments may not have been shared by older respondents.

Although we collected and analyzed respondents' information on age, gender, and region at different time periods of COVID-19, this study still has some limitations because the psychosocial and behavioral responses may be affected by many other factors that were not under our control. For example, the governmental prevention and control managements might differ during each wave of the pandemic, which made it difficult to quantify these actions in analyses. In addition, the overall survey process was based on a cellphone application so that the population selection bias was unavoidable among the respondents. For example, the elderly peoples who were not familiar with the smartphone or the people who were under-educated were less likely to complete this survey. Furthermore, the COVID-19 survey and the SARS survey were two different surveys that do not ensure comparability. However, the changing trend in respondents' psychosocial behaviors still can provide some useful information.

Although the SARS and COVID-19 occurred 17 years apart, the results of these two surveys had a similar trend, which showed that the degree of attention, personal protection, and psychosocial impact all fit a bell curve shape: the score was lowest at the initial outbreak, peaked when new cases were exponentially growing, and then decreased when the disease was under control. This finding suggested that we should not focus only on the biological bell curve of epidemics, but also pay attention to its psychologic, cognitive, and preventive measure curves. We all need to recognize this natural phenomenon and our public policy preparedness must take this into account and attempt to move the social/psychological curve to the left in order to minimize and flatten the biologic curve.

In summary, this study showed that during the outbreak of COVID-19, people in the epicenter paid more attention to the epidemic than those in other areas. Respondents had a low degree of cooperation with the prevention and control measures in the early stage, with a gradual increase as the epidemic progressed. The level of self-protection reported by people in the epicenter was always higher than that in other areas. The major perceived problems regarding the prevention and control measures were shortages of medical supplies and shortages of medical staff. The major causes of panic were an abundance of disorder and inaccurate information and shortages of PPE. The biggest difference between this new epidemic and the SARS epidemic, which took place 17 years ago, was the significant increase in the degree of cooperation with control measures and self-protection reported by the respondents. These data indicate that major public health events not only affect people physically but also affect their psychosocial and behavioral responses. Furthermore, these impacts may constantly change during an epidemic and may differ according to location and demographic characteristics. Results from this study provide the important information that government should increase medical supplies at the beginning of the pandemic, provide official information and block inaccurate information on time to stop rumor spreading. In addition, government also should provide psychological consulting from the beginning of the pandemic, which protect people from intervened mental health issues at their early stages.

## Data Availability Statement

The raw data supporting the conclusions of this article will be made available by the authors, without undue reservation.

## Ethics Statement

Ethical review and approval was not required for the study on human participants in accordance with the local legislation and institutional requirements. Written informed consent for participation was not required for this study in accordance with the national legislation and the institutional requirements.

## Author Contributions

YM, PY, and HZhan had the idea for the study and had full access to all of the data in the study and take responsibility for the integrity of the data and the accuracy of the data analysis. HY, XX, JH, JM, HZhan, PY, and YM drafted the paper. HY, XX, JH, HZhao, XL, XS, and SZ did the analysis. All authors critically revised the manuscript for important intellectual content and gave final approval for the version to be published.

## Conflict of Interest

The authors declare that the research was conducted in the absence of any commercial or financial relationships that could be construed as a potential conflict of interest.

## Publisher's Note

All claims expressed in this article are solely those of the authors and do not necessarily represent those of their affiliated organizations, or those of the publisher, the editors and the reviewers. Any product that may be evaluated in this article, or claim that may be made by its manufacturer, is not guaranteed or endorsed by the publisher.
