# Supplementary material for: Public Psychosocial and Behavioral Responses in the First Wave of COVID-19 Pandemic: A Large Survey in China
Source: Front Psychiatry. 2021 Jul 28;12:676914. doi: 10.3389/fpsyt.2021.676914 (PMC8355736; doi:10.3389/fpsyt.2021.676914)
Supplement: Supplementary file 1 [file Table_1.DOCX]

**Supplemental table**

**Supplemental Table 1. Part of the international publications using/citing the data from Horizon iDataWay**

|  | **Title** | **Time** | **Media / platform** |
| --- | --- | --- | --- |
| 1 | Chinese Government Increasingly Interested in Public Opinion Data | 2016-12-05 | US The official website of the Chicago Regional Radio  https://www.wbez.org/shows/worldview/chinese-government-increasingly-interested-in-public-opinion-data/fc754ad2-e6dc-4dda-9b28-59eee23d6f9c/amp |
| 2 | These Lucky Chinese Citizens Get to Vote—on How Their City Spends Its Money | 2018-06-02 | US "Wall Street Journal" website  https://www.wsj.com/articles/a-chinese-city-puts-it-to-a-vote-how-should-we-spend-our-money-1527845402 |
| 3 | Why China’s dog-meat market has expanded | 2017-07-17 | UK "The Economist" website  https://www.economist.com/the-economist-explains/2017/07/16/why-chinas-dog-meat-market-has-expanded |
| 4 | Religion in China on the Eve of the 2008 Beijing Olympics | 2008-05-01 | US well-known think tank Pew Center website  https://www.pewforum.org/2008/05/01/religion-in-china-on-the-eve-of-the-2008-beijing-olympics/ |
| 5 | What are China’s 12345 hotlines? | 2017-02-07 | UK "The Economist" website  https://www.economist.com/the-economist-explains/2017/02/07/what-are-chinas-12345-hotlines |
| 6 | Asia-Pacific Public Poll on International Issues | 2016-12-20 | Official website of the East Asian Institute, a well-known think tank in South Korea  http://www.eai.or.kr/main/english/program_list01.asp?code=58 |
| 7 | Asia in the Age of Uncertainty | 2016-12-20 | Official website of Lowy institute, an Australia well-known think tank in Australia  https://www.lowyinstitute.org/sites/default/files/documents/Asia%20in%20the%20Age%20of%20Uncertainty%2C%20Jan%202017%20CCGA%20LI%20EAI%20Dataway%20NPO%20APFC.pdf |
| 8 | Note to Trump: US and Chinese Publics Want Their Nations to Be Active in World Affairs | 2016-12-20 | US "Pass blue" website  https://www.passblue.com/2016/12/20/note-to-trump-us-and-chinese-publics-want-their-nations-to-be-active-in-world-affairs/ |
| 9 | Victor Yuan, “Public Opinion and Public Policy in China: Insights from the Dataway Horizon” | 2016-11-28 | Official website of the Chicago Global Affairs Commission  https://voices.uchicago.edu/eastasia/2016/11/28/november-29-victor-yuan-public-opinion-and-public-policy-in-china-insights-from-the-dataway-horizon/ |
| 10 | C100 Survey Reveals Hopes and Concerns of American and Chinese People for U.S.-China Relations | 2018-04-06 | US "Committee of 100" website  https://www.committee100.org/announcements/c100-survey-reveals-hopes-and-concerns-of-american-and-chinese-people-for-u-s-china-relations/ |
| 11 | Committee of 100 Releases 2017 U.S.-China Public Perceptions Survey | 2017-05-19 | US "PR newswire" website  https://www.prnewswire.com/news-releases/committee-of-100-releases-2017-us-china-public-perceptions-survey-300461023.html |
| 12 | Scholars, business leaders to explore U.S.-China cooperative opportunities at Yale | 2018-04-25 | CHN "Xinhuanet" English version website  http://www.xinhuanet.com/english/2018-04/25/c_137134321.htm |
| 13 | Japan-China Public Opinion Survey 2018 | 2018-10-11 | Official website of the Genron NPO, a well-known think tank in Japan  http://www.genron-npo.net/en/archives/181011.pdf |
| 14 | Why do the Japanese have negative views of China? | 2019-10-25 | Official website of the Genron NPO, a well-known think tank in Japan  http://www.genron-npo.net/en/opinion_polls/archives/5506.html |
| 15 | How Japan and China Feel about Each Other | 2018-10-24 | JP The official website of NHK TV  https://www3.nhk.or.jp/nhkworld/en/news/backstories/279/ |
| 16 | Poll Shows Over 40% of Chinese Have a Favorable View of Japan | 2018-11-08 | JP "Nippon" offcial website  https://www.nippon.com/en/features/h00322/poll-shows-over-40-of-chinese-have-a-favorable-view-of-japan.html |
| 17 | Japanese views on China remain negative despite thaw in ties | 2019-10-24 | JP "Kyoto News" official website  https://english.kyodonews.net/news/2019/10/5d783ea2cb73-japanese-views-on-china-remain-negative-despite-thaw-in-ties.html?phrase=obon&words= |
| 18 | The growing gap in how Japan and China view each other | 2019-11-22 | JP "Tokyo Review" official website  https://www.tokyoreview.net/2019/11/the-growing-gap-in-how-japan-and-china-view-each-other/ |
| 19 | More Chinese have negative view on Japan-China relations, survey finds | 2016-09-24 | JP "Japan Times" official website  https://www.japantimes.co.jp/news/2016/09/24/national/chinese-negative-view-japan-china-relations-survey-finds/#.XoWEiVN950w |

**Supplemental Table 2. COVID-19 Survey Questions**

| Disease Awareness | 1 | What is your degree of attention to this epidemic? | Please score on a scale of 0-10, with 0 means zero attention and 10 means a lot. |
| --- | --- | --- | --- |
|  | 2 | What is your level of understanding about this epidemic? | Please score on a scale of 0-10, with 0 being poorly understood and 10 being well understood |
| Personal Behavioral Responses | 3 | Local governments have introduced prevention and control measures. What is your level of cooperation? | Please score on a scale of 0-10, with 0 being poor cooperation and 10 being very good cooperation.  Stop visiting the relatives  Avoid family gathering  Cancel travel plans  Stay home and avoid going to crowded places  Avoid taking public transport  Nothing, live as usual |
|  | 4 | What is your degree of self-protection in this epidemic? | Please score on a scale of 0-10, with 0 being poor self-protection and 10 being very good self-protection.  Wear masks in public places  Wash hands more frequently  Clean the home with disinfectant  Sufficient air ventilation in the house  Measure body temperature frequently  Exercise more frequently  Avoid eating raw food  Avoid contacting wild animals  Nothing, live as usual |
| Psychosocial Effects | 5 | Do you feel panic about this epidemic? | Please score on a scale of 0-10, with 0 means no panic and 10 means very panic. |
|  | 6 | What do you feel panic mainly? [Multiple Choice] | Options: |
|  |  |  | Too much disorderly and cluttered information from different source |
|  |  |  | Neighbors coming from other/Hubei Province |
|  |  |  | PPEs (masks) were sold out |
|  |  |  | Family members not taking the disease seriously |
|  |  |  | Being contact with people from Hubei province/I live in Wuhan (Hubei) |
|  | 7 | Which aspect does the government need to improve in their prevention and control? [Multiple Choice] | Options: |
|  |  |  | Shortages of medical staff |
|  |  |  | Uneven distribution of PPEs |
|  |  |  | Information delay and clutter |
|  |  |  | Poor tracking of high-risk personnel |
|  |  |  | Inadequate publicity of prevention and treatment plans |
|  |  |  | No effective drug against COVID-19 |
|  | 8 | Please rate the work of local regulations. | Total of 100 points |
|  | 9 | Do you believe that our country can make it through this epidemic? | Please score on a scale of 0-10, with 0 means having no confidence and 10 means very confident. |
| Basic Personal Information | 10 | Age | How old are you? |
|  | 11 | Gender | Male/Female |
|  | 12 | Region | Wuhan City/Hubei Province (non-Wuhan cities)/Other Provinces |

**Supplemental Table 3 Three COVID-19 Surveys Scoring Results**

|  |  |  | Disease Awareness | | Personal Behavioral Responses | | Psychosocial Effect | | |
| --- | --- | --- | --- | --- | --- | --- | --- | --- | --- |
|  | Region | Number of Responses | Attention | Understanding | Cooperation | Self-protection | Panic | Satisfaction | Confidence |
| Survey 1 | Overall | 1674 | 9.42±1.16 | 8.05±1.70 | 9.11±1.53 | 8.88±1.45 | 6.17±2.63 | 73.48±24.15 | 8.56±1.80 |
|  | Wuhan | 80 | 9.50±1.18 | 7.94±1.96 | 8.75±2.02 | 8.90±1.67 | 7.04±2.65 | 55.58±28.89 | 7.90±1.97 |
|  | Non-Hubei | 1571 | 9.42±1.17 | 8.05±1.69 | 9.14±1.48 | 8.88±1.44 | 6.13±2.62 | 74.47±23.53 | 8.60±1.77 |
|  | P-value |  | 0.547 | 0.565 | **0.025** | 0.891 | **0.002** | **<0.001** | **0.001** |
| Survey 2 | Overall | 6685 | 9.50±1.24 | 8.49±1.55 | 9.34±1.34 | 9.02±1.33 | 6.11±2.66 | 81.57±18.22 | 9.18±1.43 |
|  | Wuhan | 252 | 9.77±0.92 | 8.65±1.47 | 9.30±1.65 | 9.25±1.25 | 6.95±2.41 | 62.68±23.03 | 8.68±1.89 |
|  | Non-Hubei | 6036 | 9.49±1.25 | 8.47±1.55 | 9.34±1.32 | 9.00±1.33 | 6.09±2.65 | 82.62±17.33 | 9.20±1.40 |
|  | P-value |  | **<0.001** | 0.072 | 0.661 | **0.003** | **<0.001** | **<0.001** | **<0.001** |

| Survey 3 | Overall | 4855 | 9.14±1.44 | 8.23±1.69 | 9.28±1.30 | 9.00±1.41 | 6.25±2.73 | 87.28±15.25 | 9.36±1.23 |
| --- | --- | --- | --- | --- | --- | --- | --- | --- | --- |
|  | Wuhan | 221 | 9.48±1.32 | 8.40±1.85 | 9.36±1.33 | 9.14±1.55 | 6.65±2.92 | 74.93±20.32 | 9.13±1.55 |
|  | Non-Hubei | 4351 | 9.12±1.45 | 8.19±1.70 | 9.29±1.30 | 9.00±1.41 | 6.16±2.72 | 88.01±14.65 | 9.38±1.21 |
|  | P-value |  | **<0.001** | 0.083 | 0.421 | 0.158 | **0.009** | **<0.001** | **0.003** |
